# Deep Learning in Optical Coherence Tomography Angiography: Current Progress, Challenges, and Future Directions

**DOI:** 10.3390/diagnostics13020326

**Published:** 2023-01-16

**Authors:** Dawei Yang, An Ran Ran, Truong X. Nguyen, Timothy P. H. Lin, Hao Chen, Timothy Y. Y. Lai, Clement C. Tham, Carol Y. Cheung

**Affiliations:** 1 Department of Ophthalmology and Visual Sciences, The Chinese University of Hong Kong, Hong Kong SAR, China; 2 Hong Kong Eye Hospital, Hong Kong SAR, China; 3 Department of Computer Science and Engineering, The Hong Kong University of Science and Technology, Hong Kong SAR, China; 4 2010 Retina & Macula Centre, Hong Kong SAR, China

**Keywords:** optical coherence tomography angiography, image quality, artificial intelligence, deep learning, medical image analysis, diabetic macular ischemia, diabetic retinopathy, retinal vascular diseases, glaucoma

## Abstract

Optical coherence tomography angiography (OCT-A) provides depth-resolved visualization of the retinal microvasculature without intravenous dye injection. It facilitates investigations of various retinal vascular diseases and glaucoma by assessment of qualitative and quantitative microvascular changes in the different retinal layers and radial peripapillary layer non-invasively, individually, and efficiently. Deep learning (DL), a subset of artificial intelligence (AI) based on deep neural networks, has been applied in OCT-A image analysis in recent years and achieved good performance for different tasks, such as image quality control, segmentation, and classification. DL technologies have further facilitated the potential implementation of OCT-A in eye clinics in an automated and efficient manner and enhanced its clinical values for detecting and evaluating various vascular retinopathies. Nevertheless, the deployment of this combination in real-world clinics is still in the “proof-of-concept” stage due to several limitations, such as small training sample size, lack of standardized data preprocessing, insufficient testing in external datasets, and absence of standardized results interpretation. In this review, we introduce the existing applications of DL in OCT-A, summarize the potential challenges of the clinical deployment, and discuss future research directions.

## 1. Introduction

**Figure 1:**
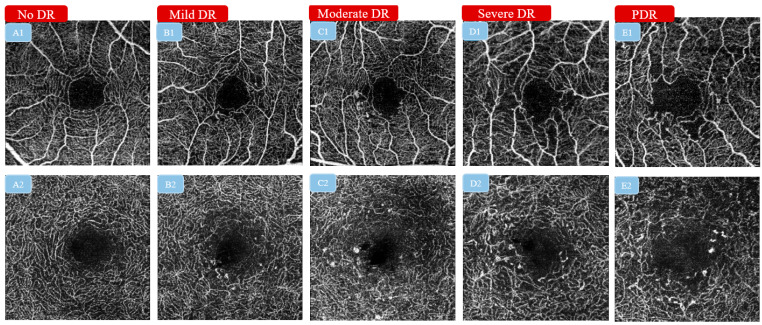
Examples of a series of 3 × 3 mm^2^ superficial capillary plexus (SCP) and deep capillary plexus (DCP) optical coherence tomography angiography (OCT-A) images illustrating different severities of diabetic retinopathy. (**A1**–**E1**): SCP OCT-A images illustrating the alteration of the FAZ area and the surrounding vasculature from no DR to PDR. (**A2**–**E2**): DCP OCT-A images illustrating the alteration of the FAZ area and the surrounding vasculature from no DR to PDR. OCT-A: optical coherence tomography angiography; SCP: superficial capillary plexus; DCP: deep capillary plexus; FAZ: foveal avascular zone; DR: diabetic retinopathy; PDR: proliferate diabetic retinopathy.

Optical coherence tomography angiography (OCT-A), as the functional extension of structural optical coherence tomography (OCT), is a novel imaging modality that can provide high-resolution and depth-resolved angiographic flow images by utilizing motion contrast [1]. With the advantages of being non-invasive and more readily available, OCT-A has opened a wealth of possibilities for investigating different types of vascular damage, such as diabetic retinopathy (DR) [2], age-related macular degeneration (AMD) [3], glaucoma [4], and retinal vein occlusion (RVO) [5], as it enables the assessment of microvasculature alterations in different vascular plexuses of the retina and optic nerve head. Taking DR as an example, previous studies have validated OCT-A as an alternative to fluorescein angiography (FA) for the assessment of DR-related pathological features, such as microaneurysms, capillary non-perfusion, and neovascularization [6]. Furthermore, quantitative OCT-A metrics, such as vessel density and foveal avascular zone (FAZ) area, have been correlated with the severity of DR (Figure 1) and visual acuity (VA) [7,8,9]. Longitudinal studies found that these quantitative metrics can help to predict DR progression and diabetic macular edema (DME) development [10,11]. With its reliable capacity for disease detection and prediction, the uptake of OCT-A in clinics has been sustainably growing.

Recently, research in OCT-A has been evolving in tandem with tremendous improvement in image analysis driven by artificial intelligence (AI), particularly by deep learning (DL) [12,13], a subfield of machine learning (ML), which is based on deep neural networks (DNNs) with multiple processing layers to learn the feature representations with multiple levels of abstraction in images [14,15]. With the availability of the large amount of high-quality images, ophthalmology is especially well positioned to attain the benefits of advances in DL, and has subsequently become a vital driving force behind the various application of DL frameworks [16,17]. Notably, DL algorithms based on retinal photographs and OCT images have made important breakthroughs, such as those in DR [18,19], AMD [20], and glaucoma [21]. Although the implementation of OCT-A with DL is gaining attention, it is still relatively scarce. How the combination of advanced DL approaches could further enhance the clinical utility of OCT-A and optimize the clinical workflow remains as an open question.

This review summarizes recent studies on DL-based OCT-A image analysis (Table 1), discusses the potential challenges of the clinical deployment, and proposes future research directions.

## 2. Deep Learning-Based Algorithms for OCT-A Image Quality Control

### 2.1. Image Quality Grading

**Figure 2:**
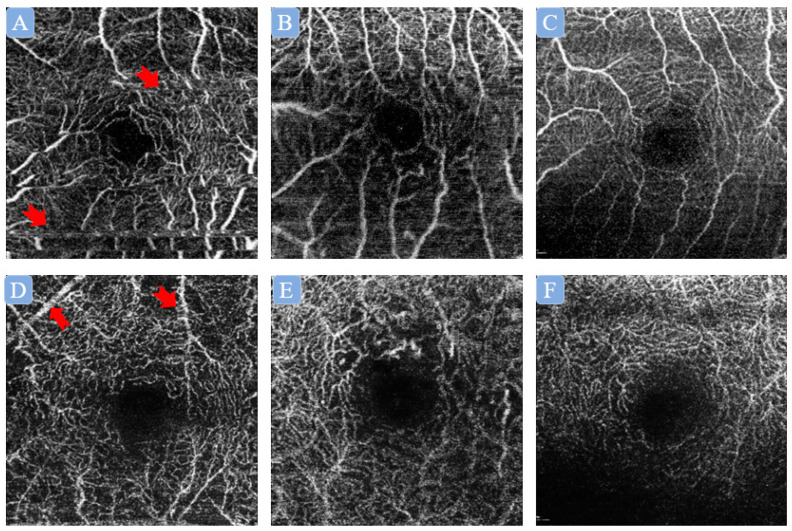
Illustration of different kinds of artifacts in superficial capillary plexus (SCP) and deep capillary plexus (DCP) optical coherence tomography angiography (OCT-A) images. (**A**–**C**) Artifacts in SCP OCT-A images, namely, (**A**) movement artifact (red arrow); (**B**) defocus artifact; (**C**) shadow artifact. (**D**–**F**) Artifacts in DCP OCT-A images, namely, (**D**) projection artifact (red arrow); (**E**) defocus artifact; (**F**) shadow artifact.

The generation of artifacts during image acquisition is an inherent challenge for any clinical imaging modalities, including OCT-A. There are different types of artifacts present in OCT-A images (Figure 2), and the presence of artifacts could impede image interpretation both qualitatively and quantitatively [22,23]. In most previous studies, image quality grading was performed manually. However, it is a labor-intensive, time-consuming, and resource-demanding task, which has been a significant limitation and barrier to the application of OCT-A in clinical settings. Notably, current research has shown the promise of DL-based automated image quality assessment. For example, Lauermann et al. [24] developed a multilayer convolutional neural network (CNN) for classifying foveal-center 3 × 3 mm^2^ superficial capillary plexus (SCP) OCT-A images as either sufficient or insufficient quality. The developed network was trained by a total of 160 SCP OCT-A images (sufficient group: 80; insufficient group: 80) and tested on 40 unseen images. The proposed network attained a training accuracy of 97% and validation accuracy of 100% for classifying the images into the binary classification. Yang et al. [25] developed a multitask DL network to assess both 3 × 3 mm^2^ SCP and deep capillary plexus (DCP) OCT-A images. By using more than 3500 SCP and DCP OCT-A images, respectively, for training, and another 480 SCP and DCP OCT-A images, respectively, for testing, they reported the DL network achieved areas under the receiver operating characteristic curves (AUROCs) above 0.982 for the gradability task, and AUROCs above 0.973 for the measurability task for both SCP and DCP OCT-A images derived from two types of OCT-A devices. Likewise, in order to fulfill the need for selecting qualified images for different settings, Dhodapkar et al. [26] trained two separate networks based on 8 × 8 mm^2^ SCP OCT-A images to classify high-quality images, which were for research use, and low-quality images, which should be excluded. They reported AUROCs above 0.97 for both networks. Remarkably, the developed networks were further tested on 6 × 6 mm^2^ SCP OCT-A images with good results (AUROCs > 0.85).

### 2.2. Image Reconstruction

**Figure 3:**
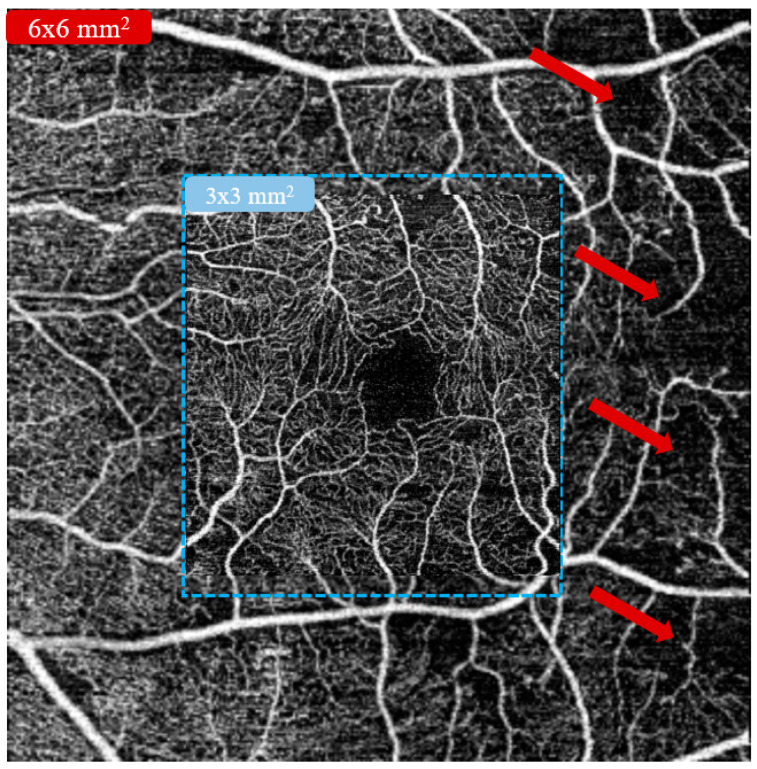
An example illustrating that the 6 × 6 mm^2^ optical coherence tomography angiography (OCT-A) scanning protocol with larger field of view can detect non-perfusion area (red arrow) outside of the standard centralized 3 × 3 mm^2^ scanning protocol.

The 3 × 3 mm^2^ scan is the most commonly used scanning protocol in recent OCT-A studies as it preserves a higher resolution than other wider field scans (e.g., 6 × 6 mm^2^ scan) for studying microvascular changes. However, the interpretation of microvascular alteration should not be limited to a 3 × 3 mm^2^ area as the pathological changes can also manifest elsewhere (Figure 3) [27]. Therefore, it is conceivable that both wider field of view and higher resolution should be well incorporated to better address the needs of the clinical assessment. Notably, Gao et al. [28] proposed a DL-based angiogram reconstruction network for reconstructing low-resolution 6 × 6 mm^2^ superficial vascular complex (SVC) OCT-A images. In the experiment, they reported that the reconstructed images presented reduced noise and enhanced connectivity when compared to the original ones. They also concluded that the proposed network did not generate false flow signal at realistic noise intensities during image reconstruction. Later on, they further developed another reconstruction network for 6 × 6 mm^2^ intermediate capillary plexus (ICP) and DCP OCT-A images [29]. Their results indicated that the reconstruction network also applied well to the ICP and DCP as the newly developed model significantly reduced noise intensity and improved vascular connectivity without generating false flow signal. Zhang et al. [30] proposed a frequency-aware inverse-consistent generative adversarial network to improve the resolution of 6 × 6 mm^2^ SCP OCT-A images by using unpaired 3 × 3 mm^2^ and 6 × 6 mm^2^ images. By enabling the frequency transformations to refine the high-frequency information while retaining low-frequency information, their model successfully reconstructed the OCT-A images and outperformed other state-of-the-art methods in terms of peak signal-to-noise ratio (PSNR), structural similarity index measure (SSIM), and normalized mutual information (NMI).

## 3. Deep Learning-Based Algorithms for OCT-A Image Segmentation

### 3.1. Foveal Avascular Zone Area

The FAZ is a region surrounding the fovea which is devoid of retinal capillaries. It has been one of the most reported metrics ever since the invention of OCT-A. The literature has related the size and the intactness of the FAZ to the severity and progression of many retinal diseases [9], as well as the deterioration of VA [31,32]. Since both the shape and intactness of the FAZ carry important clinical implications, many studies have developed algorithms for automated segmentation and measurement. For instance, by using eighty 1 × 1 mm^2^ OCT-A images from healthy volunteers, Prentašic et al. [33] trained and validated a DL network for segmenting FAZ area, attaining a maximum mean accuracy of 0.83. Mirshahi et al. [34] also developed a DL network for the segmentation and measurement of the FAZ. Specifically, for the FAZ segmentation task, the proposed network achieved a mean dice similarity coefficient (DSC) of 0.94 ± 0.04 when compared to the results produced by the device’s built-in software, while for the FAZ measurement task, among the healthy subjects, excellent agreements were reported between the device-based and manual measurement (95% limits of agreement (LoAs) of −0.005 to 0.026 mm^2^) as well as between the DL and manual measurements (95% LoAs of 0.000 to 0.009 mm^2^). Similarly, Guo et al. [35] also proposed a DL network with an encoder–decoder architecture to automatically perform the segmentation and quantification of the FAZ area in SCP under different brightness/contrast settings. They reported a maximum mean DSC of 0.976 ± 0.01 when comparing the automatic segmentation results against the ground truth, and a correlation coefficient of 0.997 between ground truth and automatic segmentation results for calculating the FAZ area.

### 3.2. Vessel Segmentation

Retinal vasculature is critical for the nourishment of retinal tissue to maintain the normal function of the visual pathway. Pathological alterations in vascular structure have not only been used for the detection and classification of different fundus diseases [36], but also been linked with systemic diseases [37]. Studies have implemented OCT-A with DL to facilitate an automatic vessel segmentation. Ma et al. [38] introduced a novel split-based coarse-to-fine method for vessel segmentation in both SVC and deep vascular complex (DVC) OCT-A images. The network consisted of a segmentation module to first produce the preliminary confidence vessel map, and then further used a consecutive refining model to refine and optimize the contour of the microvasculature. The model outperformed both traditional and other state-of-the-art DL models by achieving the highest AUROC, accuracy, kappa score, and dice coefficient for segmenting the vessel. Liu et al. [39] proposed a disentangled representation DL model to facilitate the vessel segmentation across different OCT-A devices. By enabling the DL model to learn the disentanglement of the anatomical component (the microvasculature in images) and the local contrast component (the image background noise diversities among different OCT-A devices), their model demonstrated good performance for OCT-A vessel segmentation among different devices. Furthermore, Guo et al. [40] proposed a 3D CNN to segment vessels in SVP, ICP, and DCP directly from the angiographic volumetric data. Notably, their model was able to convert the data from three dimensions to two dimensions by using a custom projection module for connecting both the retinal layer segmentation and vasculature segmentation modules. The network achieved F1 score > 0.90 in SVP, >0.70 in ICP, and >0.78 in DCP for the vessel segmentation.

### 3.3. Non-Perfusion Area

Non-perfusion area (NPA) is a quantitative biomarker useful for characterizing retinal ischemia. The severity of retinal ischemia has been reported to not only impact anatomic and functional outcomes [7], but also associate with the clinical course and responsiveness to treatment [41,42]. With the attempt to facilitate and compare the efficiency of automated detection of NPA, Nagasato et al. [43] conducted research to compare the diagnostic ability among the DNN, support vector machines (SVMs), and seven ophthalmologists for distinguishing RVO with NPA from normal controls in both SCP and DCP OCT-A images. They reported that the performance of the DNN was significantly better than that of SVMs in mean AUROC, sensitivity, specificity, and average required time (all *p* < 0.01), as well as outperformed ophthalmologists in terms of AUROC and specificity (all *p* < 0.01). Guo et al. [44] applied a DL-based algorithm for the detection and quantification of NPA across eyes of healthy subjects compared to patients with different DR severities on widefield OCT-A images constructed by a montage of nasal, macular, and temporal scans. The algorithm showed good agreement with manual delineation (average F1 score > 0.78) for NPA segmentation across all scans. In addition, they demonstrated that NPA measured in the montage widefield images correlated with both VA and DR severity significantly, and its diagnostic accuracies for distinguishing any DR, referable DR, and severe DR were even superior to NPA measured from the traditional macular scan (all *p* < 0.0001).

### 3.4. Neovascularization

The accurate identification and segmentation of choroidal neovascularization (CNV) are essential for the diagnosis and management of chorioretinopathies [45], such as exudative AMD and myopic CNV, as they require urgent referral for timely intervention. Wang et al. [46] developed a fully automated algorithm for the detection and segmentation of CNV in OCT-A images, with a total of 1676 scan including follow-up scans used for training and testing. During testing, their algorithm attained a 100% sensitivity and 95% specificity for differentiating CNV cases from the non-CNV controls. Additionally, an intersection over union (IoU) of 0.88 was reported between the human graders and the algorithm for the segmenting the CNV membrane. Likewise, Thakoor et al. [47] developed a hybrid 3D (OCT-A volume scans)–2D (OCT-B scans) CNN to facilitate a multiclass categorical AMD classification with a total of 346 eyes (97 non-AMD, 169 non-neovascular AMD, and 80 neovascular AMD) enrolled for training, validation, and testing. They reported an accuracy up to 77.8% for classifying different stages of AMD, illustrating the tremendous potential of DL algorithms concatenating multiple imaging modalities to expedite the screening for early- and late-stage AMD patients.

## 4. Deep Learning-Based Algorithms for OCT-A Image Classification

### 4.1. The Classification of Artery and Vein

The differentiation of artery–vein (AV) analysis can not only provide valuable information for retinal diseases, but also new insights on systemic diseases. For example, the narrowing of retinal arteriole has been reported to associate with hypertension, while both arteriolar and venular tortuosity have been shown to relate to DR progression [48,49,50,51,52]. As OCT-A images are extensively reported to reveal subtle microvascular changes, recent studies have combined both DL and OCT-A to facilitate the AV classification. For example, Alam et al. [53] developed a fully CNN based on modified U-shaped architecture to differentiate arteries and veins in OCT-A images, with a transfer learning process also being integrated to compensate for a limited dataset. They reported their algorithm achieved an average accuracy of 86.75%, a mean IoU of 70.72%, and an F1 score of 82.81% on the test data, of which outperformed other state-of-the-art models, as well as the model without using transfer learning. Gao et al. [54] further proposed a CNN for AV classification in montaged widefield OCT-A images. Specifically, they used only 6 × 6 mm^2^ OCT-A images from the nasal, macula, and temporal area for training and validating the algorithm, and testing on both 6 × 6 mm^2^ and 9 × 9 mm^2^ images. The proposed algorithm attained an F1 score > 94.1% and IoU > 89.2% for the AV classification across two devices and different scan sizes.

### 4.2. The Classification of Diabetic Retinopathy Severity

In addition to retinal photograph-based algorithms, DL has also been making remarkable breakthroughs with several OCT-A-based algorithms also being built to enhance DR classification and management. For example, by enlisting diabetic eyes ranging from no DR to different severities of DR, Ryu et al. [55] developed a fully automated classification algorithm to identify the onset and referable status of DR in OCT-A images. The proposed algorithms achieved AUROCs above 0.93, and accuracies, sensitivities, and specificities all above 85% for detecting the onset of DR and referable DR, both in the internal validation and external testing. Le et al. [56] developed a CNN implemented with a transfer learning process for retraining the model to perform a trinary classification, namely, healthy, diabetic mellitus (DM) but with the absence of DR, and with the presence of DR, on OCT-A images. Their model also attained good performance with a cross-validation accuracy of 87.27%, sensitively of 83.76%, and specificity of 90.82% for differentiating the trinary outcomes, and AUROCs all above 0.97 across the binary classification among healthy, DM but with the absence of DR, and with the presence of DR. In order to utilize information both from OCT and OCT-A, Zang et al. [57] developed an automated model to produce three classification levels to facilitate the clinical diagnosis of different stages of DR. The first level of the model was designed to classify non-referable and referable DR; the second level was to differentiate no DR, non-proliferative DR (NPDR), and proliferative DR (PDR); and the third level was to perform a full DR classification, namely no DR, mild and moderate NPDR, severe NPDR, and PDR. They reported overall classification accuracies of 95.7%, 85.0%, and 71.0%, respectively, for the three classification levels.

### 4.3. The Classification of the Presence or Absence of Diabetic Macular Ischemia

**Figure 4:**
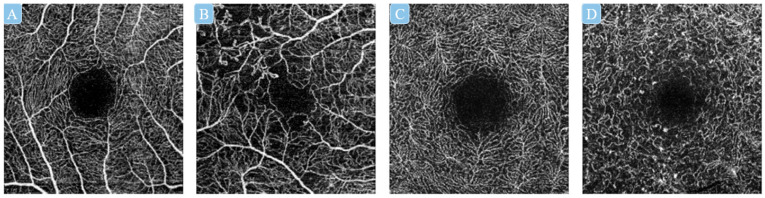
Examples of optical coherence tomography angiography (OCT-A) images in classifications of diabetic macular ischemia (DMI) on superficial capillary plexus (SCP) and deep capillary plexus (DCP). DMI classification from left to right: no DMI on SCP (**A**), DMI on SCP (**B**), no DMI on DCP (**C**), DMI on DCP (**D**). OCT-A: optical coherence tomography angiography; SCP: superficial capillary plexus; DCP: deep capillary plexus; DMI: diabetic macular ischemia.

Previous studies have reported that the severity of macular ischemia is associated with irreversible visual deterioration, as well as the treatment response following anti-VEGF therapy in eyes with concomitant DME [42]. Yang et al. [25] developed a multitask DL system to first assess the image quality, and then classify the presence or absence of diabetic macular ischemia (DMI) in both SCP and DCP OCT-A images. In order to train the model to perform “DMI assessment” based on the ETDRS protocols, they defined the presence of DMI as OCT-A images exhibiting disruption of FAZ and/or additional areas of capillary nonperfusion in the macula, while the absence of DMI was classified as images exhibiting intact FAZ outline and normal distribution of vasculature (Figure 4). Their model achieved AUROCs > 0.939 and areas under the precision–recall curves (AUPRCs) > 0.899 for the DMI assessment across three external validation datasets compromising two different types of OCT-A devices [58].

### 4.4. The Classification of Healthy Eyes and Glaucoma

Glaucoma is among the leading causes of irreversible vision loss globally. Earlier OCT-A studies have revealed that in comparison to healthy eyes, glaucoma eyes showed significant attenuations in optic disc perfusion and peripapillary vessel density [59,60]. These alterations were not only associated with worse structural and functional glaucomatous measurements, but also provide predictive values for glaucoma progression [4,60]. Of note, Bowd et al. has compared a CNN to conventional ML, i.e., gradient-boosting classifiers (GBCs), for classifying healthy and glaucomatous eyes based on OCT-A metrics [61]. Specifically, the DL model was trained and tested on 4.5 × 4.5 mm^2^ radial peripapillary capillary OCT-A optic nerve head (ONH) images, and further compared with separate GBC models trained and tested on standard OCT-A and OCT measurements. The adjusted AUPRC for classifying healthy and glaucoma eyes was significantly improved by using the DL-based CNN analysis of OCT-A metrics in comparison to the conventional GBC analysis (*p* ≤ 0.01). On the other hand, instead of comparing DL and ML models, Schottenhamml et al. [62] demonstrated that by training the CNN using 3 × 3 mm^2^ OCT-A images of different retinal projections (of the whole retina, SVC, ICP, and DCP), the CNN performed similarly well to, or even better than, the handcrafted methods for distinguishing glaucomatous eyes from healthy controls, especially when using features from the whole retina projection and the SVC projection.

## 5. Discussion

Ever since OCT-A was introduced in 2016, there has been a huge surge in literature and evidence indicating how OCT-A metrics might assist the diagnosis and staging for several retinal vascular diseases and yield prognostic values for disease progression and treatment response. However, with the complexity of the 3D information captured by OCT-A with the growing size of datasets, manual delineation, classification, and analysis can be a time-consuming and labor-intensive task and therefore make the widespread application of OCT-A in clinics rather infeasible. In recent years, DL-based models have been implemented with OCT-A to further fulfill its potential in image quality control, segmentation, and classification. Nevertheless, it should be noted that almost all studies were still in the “proof-of-concept” stage without being evaluated in real-world settings. In addition, there are substantial issues in both clinical and technical domains that should be further considered and evaluated before using OCT-A-based models in real-world clinical settings.

From the clinical perspective:

1. The nomenclature of OCT-A metrics should be further standardized.

Although quantitative OCT-A metrics might provide important clinical values, the diversified terms used in the current literature could be confusing and even misleading. For example, different terms were defined with the same definition, as research used perfusion density [63] and vessel density [64] to indicate the percentage of area occupied by perfused binarized vessels for quantifying the perfusion status of the retina. In addition, OCT-A metrics are not uniformly used across all devices. Taking FAZ area measurement as an example, Zeiss Angioplex offers the analysis of FAZ parameters such as circularity, size, and area, while Optovue enables the assessment differently by including FAZ area, perimeter, and AI/FD (circularity index/FD-300 (vessel density 300 μm from the fovea)) [65]. Moreover, various retinal segmentation strategies further contribute to the heterogeneity as some devices define the middle of the inner nuclear layer (e.g., Optovue), while others use the top of the inner nuclear layer (e.g., Topcon) as the boundary to segment DCP. All these above-mentioned issues make the homogenous description and comparison of OCT-A data among different devices infeasible. With more and more efforts being invested for standardizing the OCT-A nomenclature internationally [66,67], this will further facilitate the feasibility of the OCT-A-based algorithms being used in both clinics and scientific research.

2. The normal range of OCT-A metrics should be established.

Besides the standardization of the nomenclature, efforts should also be made for establishing the normal ranges for different OCT-A metrics. Although recent DL studies developed algorithms for automatically quantifying FAZ area and vessel density, such quantitative metrics could only be considered as a potential clinical endpoint when there is a reference range; otherwise, it would be baseless to claim any value that is out of range or even indicating abnormalities [42]. Additionally, setting up the normal range of OCT-A metrics could further facilitate the establishment of a severity grading scale for retinal diseases/abnormalities, such as for DR/DMI. Several studies have designed DL models to classify different severities of DR [68], or even distinguish eyes without DR from normal controls [69]. Additional studies would be more convincing if the changes in metrics, such as the decrease in vessel density, the increase in NPA, and the enlargement of FAZ area, can be normalized and quantitatively correlated with the severity of DR. With the normal range of OCT-A metrics across different devices being established, it will provide more insights on how to deploy OCT-A with DL to detect, stage, or even triage patients early for a more personalized intervention.

From the technical perspective:

1. The training sample size should be expanded to avoid biased models.

It is noticeable that most training sample sizes of the existing OCT-A-based DL models were relatively small, especially when compared to those trained with OCT or retinal fundus photographs. A diversified dataset is extremely important to avoid overfitting during model training, as well as to improve the generalizability of the algorithm to unseen datasets. Different data augmentation methods [70], such as cropping, rotation, and color jittering, have been widely used to enlarge the training sample size in previous studies. More recent studies have further used advanced DL technologies, such as transfer learning [71] and low-shot learning [72], to address this issue. In the future studies, the training sample size would be one of the important evaluation metrics for the robustness of algorithm, and new explorations in the computer science domain should be expected to overcome this issue.

2. External testing should be performed with data privacy and security being fully addressed.

To date, most of the DL models for OCT-A image analysis were trained and validated on datasets from a single center without further conducting external testing. Nevertheless, simply relying on the internal evaluation results might not provide comprehensive assessment (e.g., generalizability) of the model performance. One challenge for external testing is the lack of publicly available OCT-A data. Meanwhile, sharing data among institutions might raise privacy and data security issues. Currently, a new learning paradigm, named federated learning (FL) [73], might be used to bridge this gap. Different from traditional training methods pooling all data from multiple institutions into a central source, FL enables a central server to distribute models to be trained independently at the different institutions, while at the same time updating the global model by aggregating the characteristics (e.g., parameters and gradients) of the local models. It could be used to address the problem of data privacy and security as patients’ data will not be transferred beyond the firewalls of their own institutions.

3. Domain shift should also be handled properly among different OCT-A devices to increase model robustness.

As mentioned previously, various OCT-A devices use different imaging protocols and segmentation methods for evaluating microvasculature in different capillary plexuses. These domain shift issues impose pertinent challenges for real-world application of how to overcome the diversities in devices and standardize them into one framework in a device-agnostic manner. Recently, innovative techniques, such as domain adaptation [74], image to image translation [75], and image disentanglement [39], have been proposed to fill this gap. With more and more advanced techniques in computer science being proposed to address and alleviate the inherited variances of different devices, it can be expected that future DL algorithms will also attain robustness for OCT-A image analysis across different devices.

4. The value of using three-dimensional volumetric OCT-A scans is worth further exploration.

Most of the current DL algorithms were trained by 2D *en face* OCT-A images although OCT-A is able to provide 3D volumetric data, which contain much more information than the 2D images. Several research groups have explored the potential value for training the algorithms based on the volumetric data, and found that information hidden in the 3D images might actually provide added value to improve accuracy for segmenting features in DME [76], and classifying disease severity for DR [47]. However, it should be noted that computation time and memory might also increase tremendously when processing 3D volumetric data. Therefore, in order to further utilize the 3D data without increasing the computational cost, more sophisticated techniques, such as weakly supervised learning [77], might be used to deal with this hurdle. Future algorithms based on OCT-A volumetric scans could be expected to provide more important insights both theoretically and clinically.

5. The interpretability of the output from the DL algorithm should be further improved.

As DL models extract and learn features automatically without handcrafted feature designing, it is quite difficult to reveal and explain the decision process (i.e., “black box” issue). Many DL studies used activation maps or salient maps to generate heatmaps for highlighting the specific areas potentially related to the location of pathologies or the locations of the most significant features for identification [78]. More recently, there is a subdiscipline called explainable/interpretable AI (XAI) being set up to contend with the concept of the “black box” [79]. Future research might be conducted to further improve the interpretability of the OCT-A based DL models and evaluate whether the output can be considered as appropriate for a given imaging scenario.

## 6. Conclusions

The application of the DL-based OCT-A image analysis is both accurate and efficient regarding image quality control, segmentation, and classification. It is a very promising combination which sheds light on establishing the new and robust paradigm for staging of retinal diseases, such as an updated diabetic retinal disease (DRD) staging system. More importantly, it might facilitate routine clinical use of OCT-A for early detection, intervention, and management of retinal diseases. Future research is crucial in tackling existing challenges before real-world deployment, such as overcoming the domain shift among different devices, enhancing the interpretability and transparency of models’ outputs, as well as improving the generalizability of the models with patients’ privacy issues being fully addressed.

## 7. Literature Search

We searched databases of PubMed, Medline, Web of Science, Google Scholar, and Scopus for studies published in English up to 31 August 2022, using these keywords: “optical coherence tomography angiography”, “artificial intelligence”, “machine learning”, “deep learning”, “deep neural network”, and “convolutional neural network”. The reference lists from the selected articles were checked to obtain additional relevant articles not included in the databases.

**Table 1:** Summary of existing deep learning models with the combination of optical coherence tomography angiography.

Authors, Year	Input	Output	Datasets	Imaging Device	Model	Data Set-Up	Performance	Visualization	Generalizability Validation
Image quality grading									
Lauermann et al., 2019 [24]	3 × 3 mm^2^ SCP	Sufficient vs. Insufficient	(1) Training and validation: 80 images for both groups, respectively (2) Testing dataset: 20 images for both groups, respectively	Optovue	CNN (TensorFlow)	Pre-training+ training +testing	Training accuracy 97%, validation accuracy 100%, and cross entropy 0.12	\	\
Yang et al., 2022 [25]	3 × 3 mm^2^ SCP and DCP	Ungradable; gradable but unmeasurable; gradable and measurable	(1) Training and validation: over 3500 SCP and DCP images, respectively (2) Testing: 480 SCP and DCP images, respectively	Triton and Optovue	CNN (DenseNet)	Training + tuning + primary validation + external validation	AUROC > 0.948 and AUPRC > 0.866 for the gradability assessment, AUROC > 0.960 and AUPRC > 0.822 for the measurability assessment	CAM	Two external validation datasets
Dhodapkar et al., 2022 [26]	6 × 6 and 8 × 8 mm^2^ SCP	High quality vs. low quality	(1) Training and validation: 347 SCP scans (2) Testing: 32 SCP scans	Zeiss	CNN (ResNet152)	Training + tuning + primary validation + external validation	AUROC = 0.99 (95%CI 0.98–1.00) for low-quality image identification and AUROC = 0.97 (95%CI 0.96–0.99) for high-quality image identification	CAM	One external validation dataset
Image reconstruction									
Gao et al., 2020 [28]	3 × 3 and 6 × 6 mm^2^ SVC	Reconstructed HR images	(1) Training: 210 paired 3 × 3 and 6 × 6 mm^2^ SCP images (2) Testing: 88 paired 3 × 3 and 6 × 6 mm^2^ SCP images	Optovue	CNN (self-developed architecture)	Training + testing	Significantly lower noise intensity, stronger contrast, and better vascular connectivity than the original images	Reconstructed HR 6 × 6 mm^2^ SCP images	\
Gao et al., 2021 [29]	3 × 3 and 6 × 6 mm^2^ ICP, DCP	Reconstructed HR images	(1) Training and validation: 173 paired 3 × 3 and 6 × 6 mm^2^ ICP and DCP images (2) Testing: 101 paired 3 × 3 and 6 × 6 mm^2^ ICP and DCP images	Optovue	CNN (self-developed architecture)	Training + validation + testing	Significantly reduced noise intensity, improved vascular connectivity, and enhanced Weber contrast when compared to the original images	Reconstructed HR 6 × 6 mm^2^ ICP and DCP images	\
Zhang et al., 2022 [30]	3 × 3 mm^2^ and 6 × 6 mm^2^ SCP	Reconstructed HR images	(1) Training: 296 paired HR and LD images (2) Testing: 279 HR images	Optovue	GAN	Training + testing	Improved PSNR, SSIM, and normalized mutual information	Reconstructed HR 6 × 6 mm^2^ SCP images	\
FAZ segmentation									
Prentašic et al., 2016 [33]	1 × 1 mm^2^ images	Segmentation map	(1)Training: 2/3 out of 80 images (2) Testing: 1/3 out of 80 images	Unspecified prototype	CNN (self-developed architecture)	Three-fold cross-validation	A maximum mean accuracy of 0.83 when comparing the automated results with the manually segmented ones	FAZ segmentation map	\
Mirshahi et al., 2021 [34]	3 × 3 mm^2^ images	Segmentation map	(1) Training and validation: 126 images (2) Testing: 37 images	Optovue	CNN (ResNet50)	Training + validation + testing	A mean DSC of 0.94 ± 0.04 when compared to the results produced by the device’s built-in software	FAZ segmentation map	\
Guo et al., 2019 [35]	3 × 3 mm^2^ SCP	Segmentation map	(1) Training: 4/5 out of 405 images (2) Testing: 1/5 out of 405 images	Zeiss	CNN (U-Net)	Five-fold cross-validation	A maximum mean DSC of 0.976 ± 0.01 when comparing the automatic segmentation against the ground truth	FAZ segmentation	\
Vessel segmentation									
Ma et al., 2021 [38]	3 × 3 mm^2^ SVC, DVC angiograms	Segmentation map	(1) Training: 180 images from two datasets (2) Testing: 49 images from two datasets	Optovue	CNN (ResNet)	Training + testing	The proposed OCT-A-Net yielded better vessel segmentation performance than both traditional and other deep learning methods	Vessel segmentation map	\
Liu et al., 2022 [39]	3 × 3 mm^2^ SVP	Segmentation map	(1) Training: 330 scans for disentanglement, 207 scans for segmentation (2) Testing: 124 scans for segmentation	Optovue; Cirrus; Triton; Heidelberg	CNN (self-developed architecture)	Training + testing	The proposed mode achieved AUROC > 0.945, ACC > 0.924, kappa > 0.743, DSC > 0.788 for vessel segmentation in different validation datasets.	Vessel segmentation map	Three external validation datasets
Guo et al., 2021 [40]	2 × 2 mm^2^ volumetric scans	Segmentation map	(1) Training and validation: 76 cases (2) Testing: 12 cases	Unspecified	CNN (U-Net)	Training + validation + testing	F1 score > 90% for vessel segmentation in the SVP	Vessel segmentation map	\
Non-perfusion area segmentation									
Nagasato et al., 2019 [43]	3 × 3 mm^2^ SCP and DCP	Distribution map	A total of 144 normal controls and 174 RVO OCT-A images were included	Unspecified	CNN (VGG-16)	Eight-fold cross-validation	The mean AUROC, sensitivity, specificity, and average required time for distinguishing RVO OCT-A images with an NPA from normal OCT-A images were 0.986, 93.7%, 97.3%, and 176.9 s	CAM	\
Guo et al., 2021 [44]	6 × 6 mm^2^ volumetric scans	Distribution map	A total of 978 volumetric OCT-A scans	Optovue	CNN (U-Net)	Five-fold cross-validation	A mean standard deviation F1 score of 0.78 ± 0.05 in nasal, 0.82 ± 0.07 in macular, and 0.78 ± 0.05 in temporal scans	NPA distribution map	\
Neovascularization segmentation									
Wang et al., 2020 [46]	OCT-A volumetric scans	Segmentation map	(1) Training: 1566 scans (2) Testing: 110 scans	Optovue	CNN (self-developed architecture)	Training + testing	All CNV cases were diagnosed from non-CNV controls with 100% sensitivity and 95% specificity. The mean intersection over union of CNV membrane segmentation was as high as 0.88	Saliency map	\
Thakoor et al., 2021 [47]	OCT-B scans and OCT-A volumetric scans	Non-AMD, non-neovascular AMD, and neovascular AMD	(1) Training and validation: 277 cubes/B scan images (2) Testing: 69 cubes/B scan images	Unspecified	CNN (self-developed architecture)	Training + validation + testing	The hybrid 3D–2D CNNs achieved accuracy up to 77.8% in multiclass categorical classification of non-AMD eyes, eyes having non-neovascular AMD, and eyes having neovascular AMD	CAM	\
The classification of artery and vein									
Alam et al., 2020 [53]	6 × 6 mm^2^ OCT/OCT-A images	Artery–vein map	A total of 50 images	Optovue	CNN (U-Net)	Five-fold cross validation	The AV-Net achieved an average accuracy of 86.71% and 86.80%, respectively, for artery and vein on the test data, mean IOU was 70.72%, and F1 score was 82.81%	Artery–vein map	\
Gao et al., 2022 [54]	Montaged wide-field OCT-A	Artery–vein map	(1) Training: 240 angiograms (2) Testing: 302 angiograms	Optovue	CNN (U-Net)	Training + testing	For classification and identification of arteries, the algorithm achieved average sensitivity of 95.3%, specificity of 99.6%, F1 score of 94.2%, and IoU of 89.3%. For veins, the algorithm achieved average sensitivity of 94.4%, specificity of 99.7%, F1 score of 94.1%, and IoU of 89.2%	Artery–vein segmentation results	One external validation dataset
The classification of different DR severities									
Ryu et al., 2021 [55]	Both 3 × 3 and 6 × 6 mm^2^ SCP and DCP	DR vs. non-DR; referable DR vs. non-referable DR	(1) Training: 240 sets of images (comprising both 3 × 3 and 6 × 6 mm^2^) (2) Testing: 120 sets	Optovue	CNN (ResNet)	Training + testing	The proposed CNN classifier achieved an accuracy of 91–98%, a sensitivity of 86–97%, a specificity of 94–99%, and AUROCs of 0.919–0.976	CAM	\
Le et al., 2020 [56]	6 × 6 mm^2^ SCP and DCP	Healthy, no DR, and DR eyes	(1) Training and validation: 131 OCT-A images (2) Testing: 46 OCT-A images	Optovue	CNN (VGG-16)	Training + internal validation + external testing	The cross-validation accuracy of the retrained classifier for differentiating among healthy, no DR, and DR eyes was 87.27%, with 83.76% sensitivity and 90.82% specificity. The AUC metrics for binary classification of healthy, no DR, and DR eyes were 0.97, 0.98, and 0.97, respectively.	\	One external testing dataset
Zang et al., 2021 [57]	3 × 3 mm^2^ images	Three-level classifiers	A total of 303 eyes from 250 participants	Optovue	CNN (self-developed architecture)	Ten-fold cross-validation	The overall classification accuracies of the three levels were 95.7%, 85.0%, and 71.0%, respectively	CAM	\
The classification of the presence or absence of diabetic macular ischemia									
Yang et al., 2022 [25]	3 × 3 mm^2^ SCP and DCP	DMI vs. no DMI	(1) Training: 3307 SCP and 3135 DCP images (2) Testing: 421 SCP and 408 DCP images	Triton and Optovue	CNN (DenseNet)	Training + tuning + primary validation + external validation	For DMI detection, the DL system achieved AUROCs of 0.999 and 0.987 for SCP and DCP, respectively, in primary validation, and AUROCs > 0.939 in external datasets	CAM	Two external testing datasets
The classification of normal and glaucoma cases									
Bowd et al., 2022 [61]	4.5 × 4.5 ONH	Glaucoma vs. no glaucoma	A total of 130 eyes of 80 healthy individuals and 275 eyes of 185 glaucoma patients	Optovue	CNN (VGG-16)	Five-fold cross-validation	The adjusted AUPRC using CNN analysis of *en face* vessel density images was 0.97 (95%CI: 0.95–0.99)	\	\
Schottenhamml et al., 2021 [62]	3 × 3 mm^2^ SVC, ICP, and DCP	Glaucoma vs. no glaucoma	259 eyes of 199 subjects, 75 eyes of 74 healthy subjects, and 184 eyes of 125 glaucoma patients	Heidelberg	CNN (DenseNet and ResNet)	Five-fold cross-validation	The DL model attained AUROC of 0.967 on the SVP projection for differentiating glaucoma patients, which is comparable to the best reported values in the literature	CAM	\

ACC = accuracy; AMD = age-related macular degeneration; AUPRC = area under the precision recall curve; AUROC = area under the receiver operating characteristic curve; CAM = class activation map; CI = confidence interval; CNN = convolutional neural network; CNR = contrast-to-noise ratio; DCP = deep capillary plexus; DL = deep learning; DMI = diabetic macular ischemia; DenseNet = dense convolutional network; DR = diabetic retinopathy; DSC = dice similarity coefficient; DVC = deep vascular complex; EAA = extrafoveal avascular area; FA = fluorescein angiography; GAN = generative adversarial network; HR = high resolution; ICP = intermediate capillary plexus; IOU = intersection over union; LD = low resolution; NPA = non-perfusion area; OCT = optical coherence tomography; OCT-A = optical coherence tomography angiography; ONH = optic nerve head; PSNR = peak signal-to-noise ratio; ResNet = residual neural network; RVO = retinal vein occlusion; SCP = superficial capillary plexus; SVC = superficial vascular complex; VGG = visual geometry group.
